# Supplementary figures and images for: Negative regulation of miRNA sorting into EVs is mediated by the capacity of RBP PCBP2 to impair the SYNCRIP-dependent miRNA loading
Source: eLife. 2025 Jul 2;14:RP105017. doi: 10.7554/eLife.105017 (PMC12221297; doi:10.7554/eLife.105017)

Figure 1D

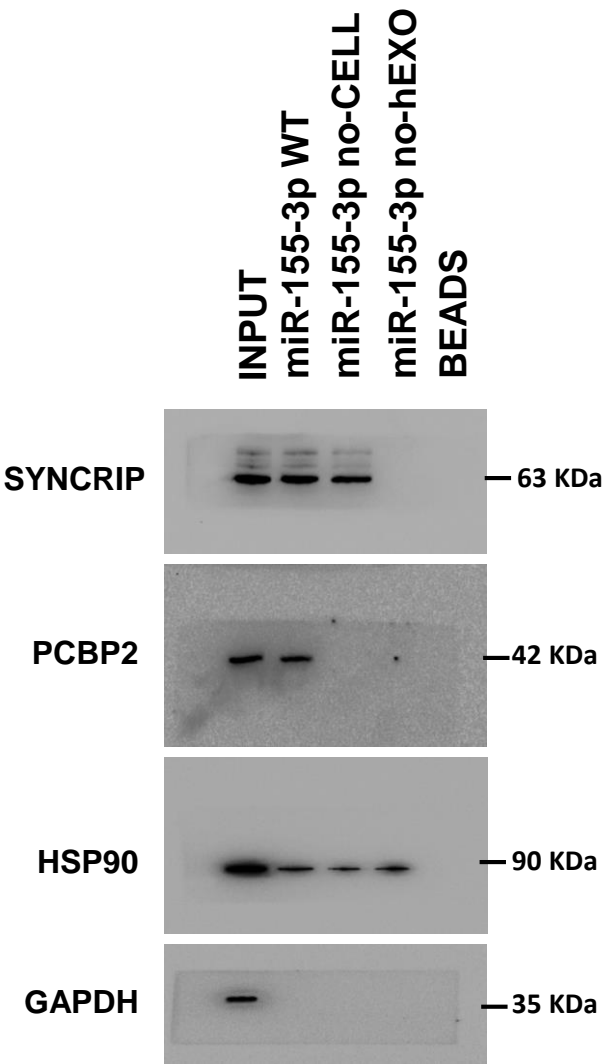

Figure 1E

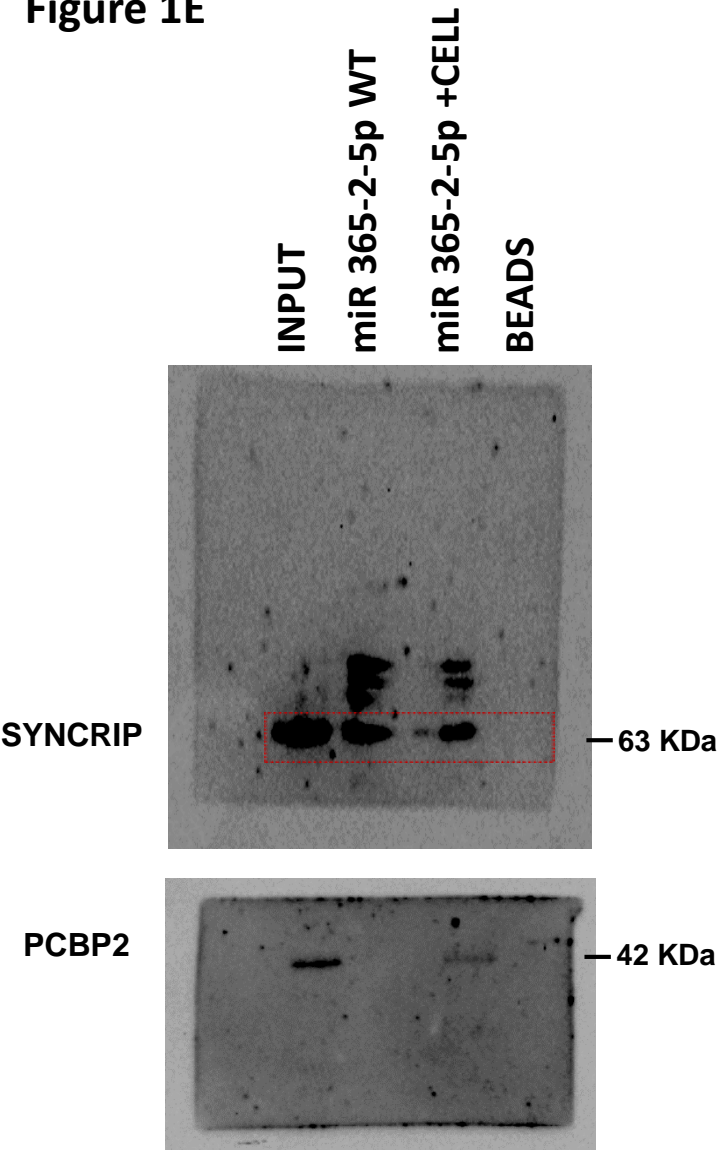

Figure 1F

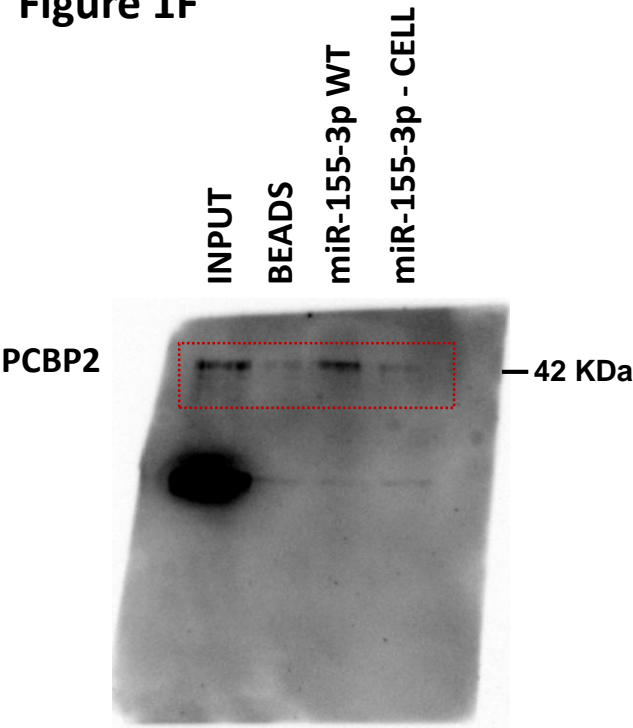

Supplement: Figure 1—source data 1. [file elife-105017-fig1-data1.pdf]

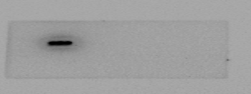

Supplement: Figure 1—source data 2. [file elife-105017-fig1-data2.zip › Figure 1D/Figure 1D GAPDH.tif]

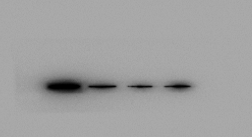

Supplement: Figure 1—source data 2. [file elife-105017-fig1-data2.zip › Figure 1D/Figure 1D HSP90.tif]

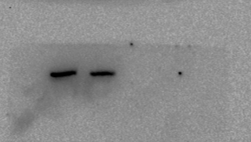

Supplement: Figure 1—source data 2. [file elife-105017-fig1-data2.zip › Figure 1D/Figure 1D PCBP2.tif]

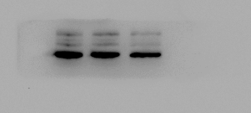

Supplement: Figure 1—source data 2. [file elife-105017-fig1-data2.zip › Figure 1D/Figure 1D SYNCRIP.tif]

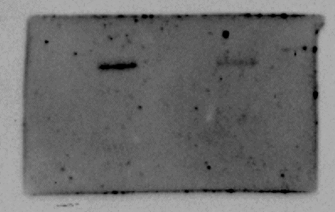

Supplement: Figure 1—source data 2. [file elife-105017-fig1-data2.zip › Figure 1E/Figure 1E PCBP2.tif]

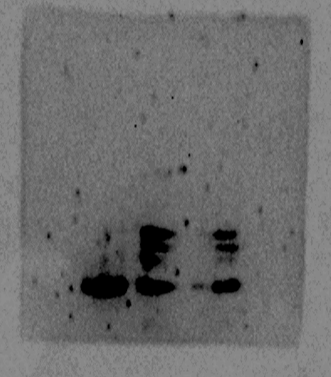

Supplement: Figure 1—source data 2. [file elife-105017-fig1-data2.zip › Figure 1E/Figure 1E SYNCRIP.tif]

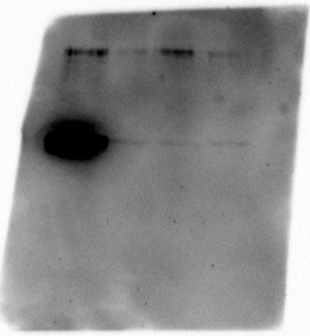

Supplement: Figure 1—source data 2. [file elife-105017-fig1-data2.zip › Figure 1F/Figure 1F PCBP2.tif]

Figure 1- figure supplement 1 B

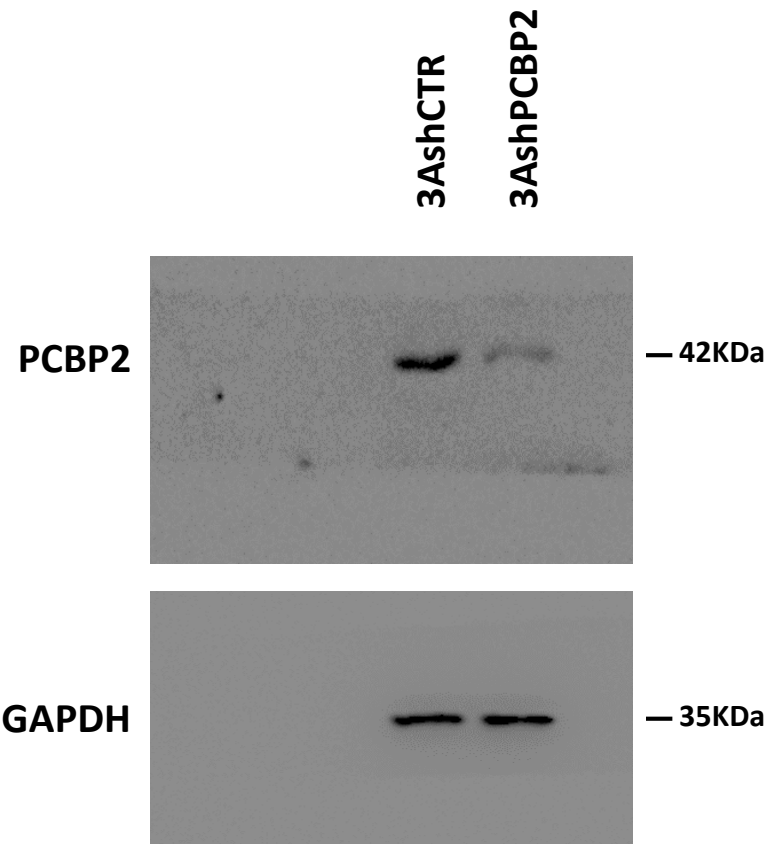

Figure 1- figure supplement 1 D

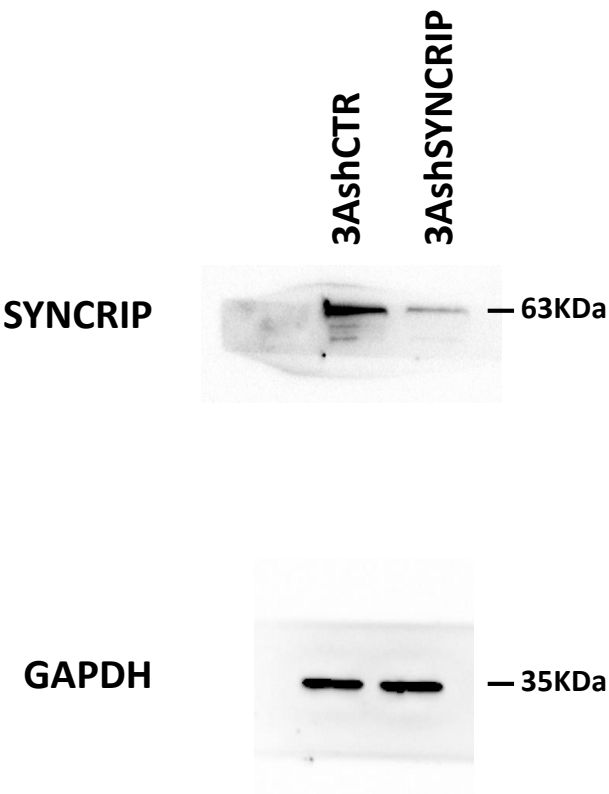

Supplement: Figure 1—figure supplement 1—source data 1. [file elife-105017-fig1-figsupp1-data1.pdf]

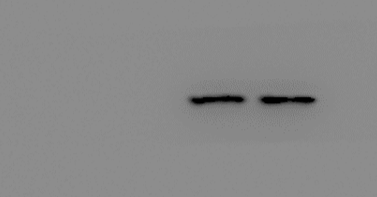

Supplement: Figure 1—figure supplement 1—source data 2. [file elife-105017-fig1-figsupp1-data2.zip › Figure 1 figure supplement 1 B/Figure 1- figure supplement 1 B GAPDH.tif]

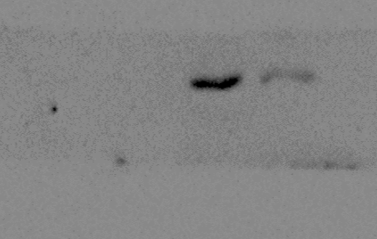

Supplement: Figure 1—figure supplement 1—source data 2. [file elife-105017-fig1-figsupp1-data2.zip › Figure 1 figure supplement 1 B/Figure 1- figure supplement 1 B PCBP2.tif]

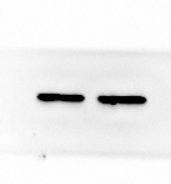

Supplement: Figure 1—figure supplement 1—source data 2. [file elife-105017-fig1-figsupp1-data2.zip › Figure 1 figure supplement 1 D/Figure 1 figure supplement 1 D GAPDH.tif]

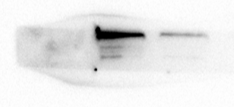

Supplement: Figure 1—figure supplement 1—source data 2. [file elife-105017-fig1-figsupp1-data2.zip › Figure 1 figure supplement 1 D/Figure 1 figure supplement 1 D SYNCRIP.tif]

Figure 1- figure supplement 2 A

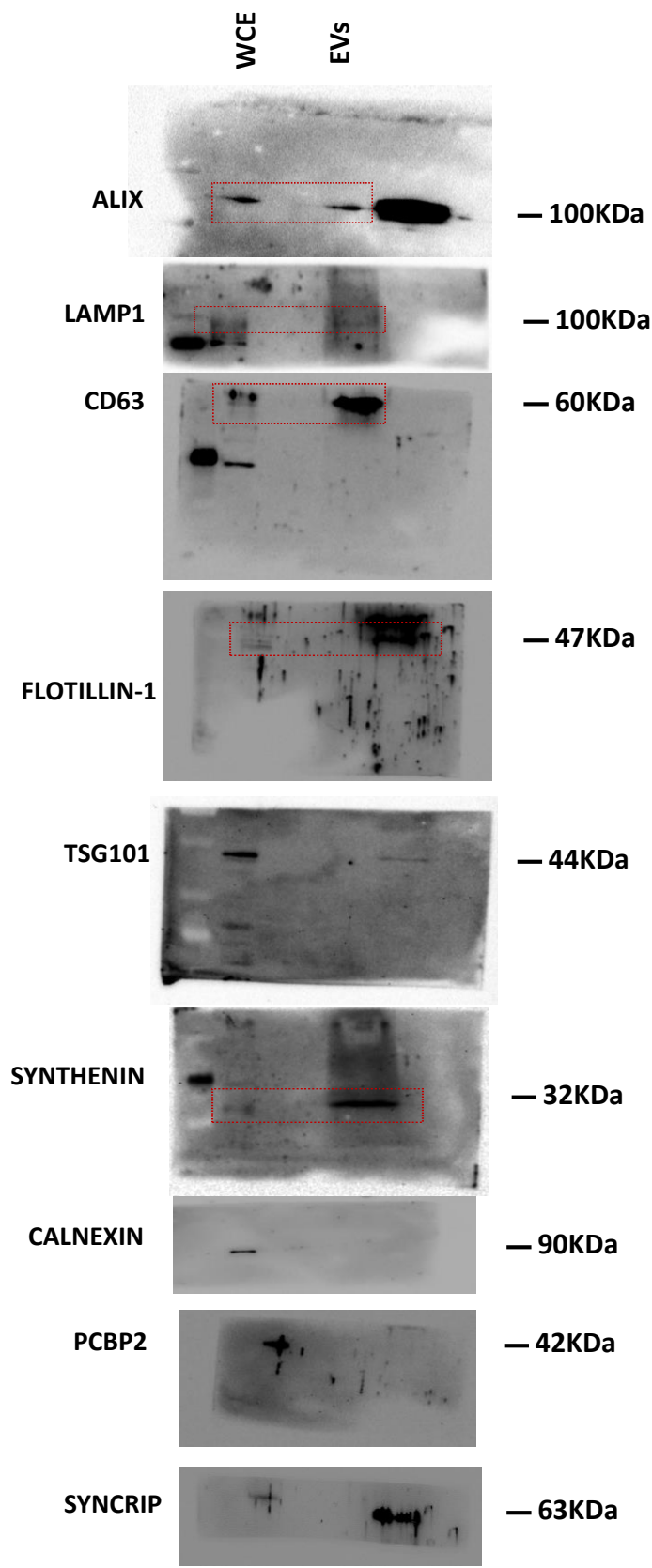

Supplement: Figure 1—figure supplement 2—source data 1. [file elife-105017-fig1-figsupp2-data1.pdf]

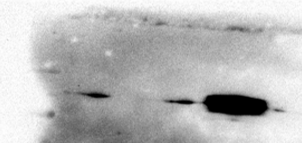

Supplement: Figure 1—figure supplement 2—source data 2. [file elife-105017-fig1-figsupp2-data2.zip › Figure 1- figure supplement 2 A/Figure 1- figure supplement 2 A Alix.tif]

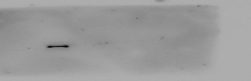

Supplement: Figure 1—figure supplement 2—source data 2. [file elife-105017-fig1-figsupp2-data2.zip › Figure 1- figure supplement 2 A/Figure 1- figure supplement 2 A CALNEXIN.tif]

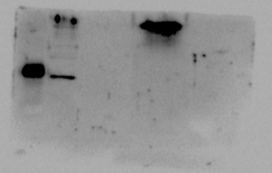

Supplement: Figure 1—figure supplement 2—source data 2. [file elife-105017-fig1-figsupp2-data2.zip › Figure 1- figure supplement 2 A/Figure 1- figure supplement 2 A CD63.tif]

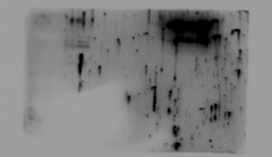

Supplement: Figure 1—figure supplement 2—source data 2. [file elife-105017-fig1-figsupp2-data2.zip › Figure 1- figure supplement 2 A/Figure 1- figure supplement 2 A Flotillin-1.tif]

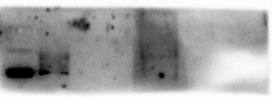

Supplement: Figure 1—figure supplement 2—source data 2. [file elife-105017-fig1-figsupp2-data2.zip › Figure 1- figure supplement 2 A/Figure 1- figure supplement 2 A LAMP 1.tif]

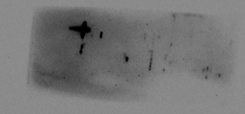

Supplement: Figure 1—figure supplement 2—source data 2. [file elife-105017-fig1-figsupp2-data2.zip › Figure 1- figure supplement 2 A/Figure 1- figure supplement 2 A PCBP2.tif]

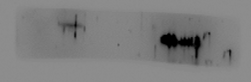

Supplement: Figure 1—figure supplement 2—source data 2. [file elife-105017-fig1-figsupp2-data2.zip › Figure 1- figure supplement 2 A/Figure 1- figure supplement 2 A SYNCRIP.tif]

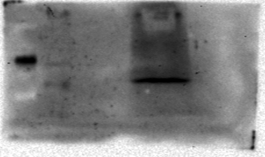

Supplement: Figure 1—figure supplement 2—source data 2. [file elife-105017-fig1-figsupp2-data2.zip › Figure 1- figure supplement 2 A/Figure 1- figure supplement 2 A SYNTHENIN.tif]

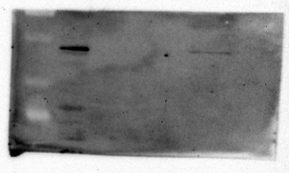

Supplement: Figure 1—figure supplement 2—source data 2. [file elife-105017-fig1-figsupp2-data2.zip › Figure 1- figure supplement 2 A/Figure 1- figure supplement 2 A TSG 101.tif]

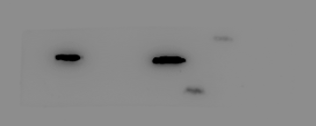

Supplement: Figure 2—source data 2. [file elife-105017-fig2-data2.zip › Figure 2A/Figure 2A GAPDH.tif]

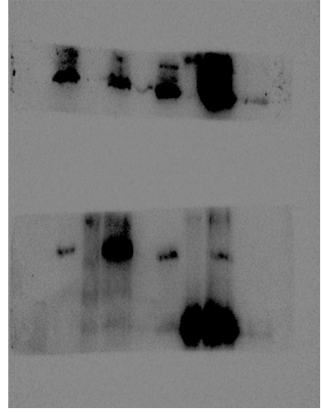

Supplement: Figure 2—source data 2. [file elife-105017-fig2-data2.zip › Figure 2A/Figure 2A SYNCRIP E PCBP2.tif]

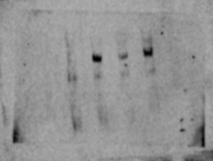

Supplement: Figure 2—source data 2. [file elife-105017-fig2-data2.zip › Figure 2B/Figure 2B EMSA.tif]

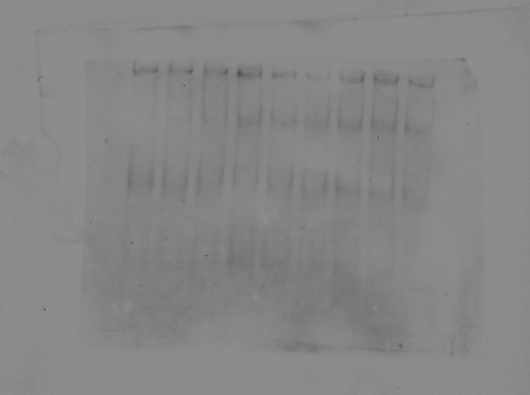

Supplement: Figure 2—source data 2. [file elife-105017-fig2-data2.zip › Figure 2C/Figure 2C EMSA.tif]

Figure 3A

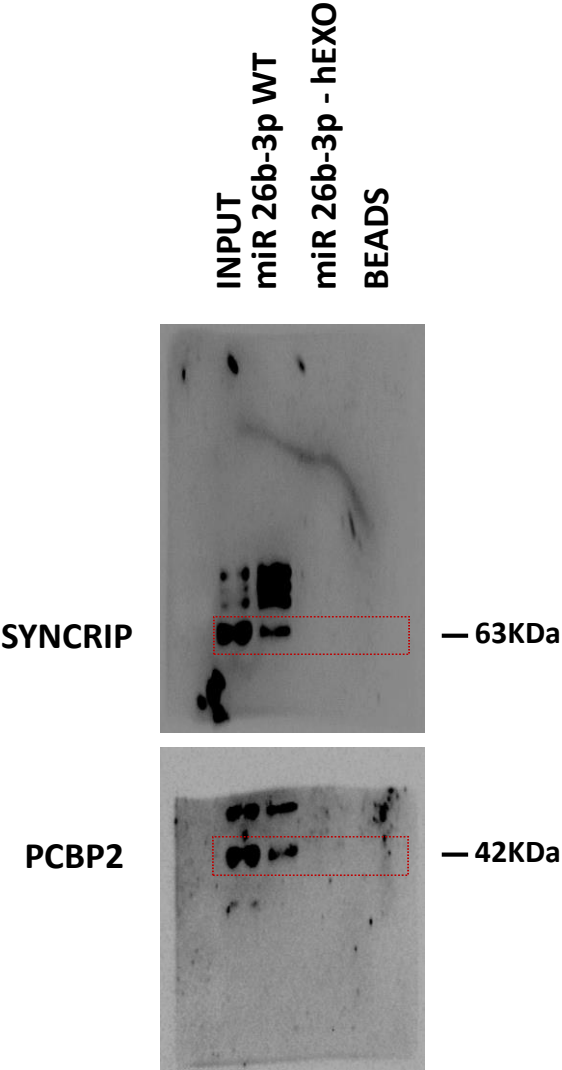

Figure 3B

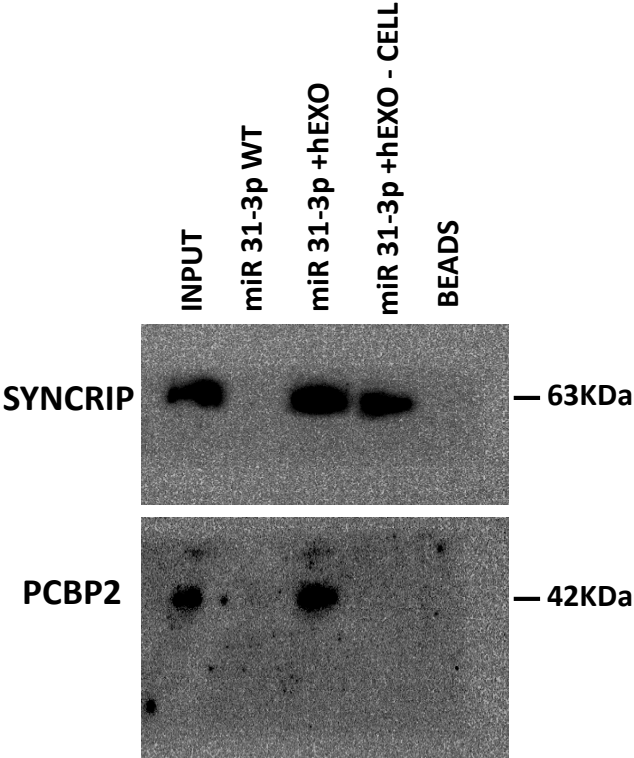

Supplement: Figure 3—source data 1. [file elife-105017-fig3-data1.pdf]

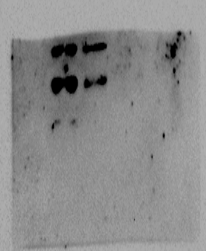

Supplement: Figure 3—source data 2. [file elife-105017-fig3-data2.zip › Figure 3A/Figure 3A PCBP2.tif]

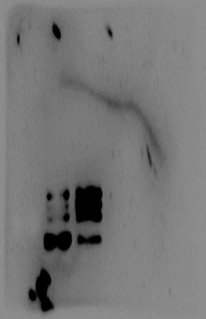

Supplement: Figure 3—source data 2. [file elife-105017-fig3-data2.zip › Figure 3A/Figure 3A SYNCRIP.tif]

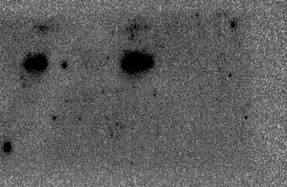

Supplement: Figure 3—source data 2. [file elife-105017-fig3-data2.zip › Figure 3B/Figure 3B PCBP2.tif]

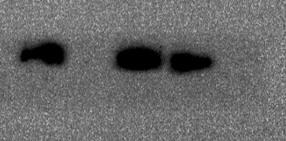

Supplement: Figure 3—source data 2. [file elife-105017-fig3-data2.zip › Figure 3B/Figure 3B SYNCRIP.tif]
