# Supplementary material for: Negative regulation of miRNA sorting into EVs is mediated by the capacity of RBP PCBP2 to impair the SYNCRIP-dependent miRNA loading
Source: eLife. 2025 Jul 2;14:RP105017. doi: 10.7554/eLife.105017 (PMC12221297; doi:10.7554/eLife.105017)
Supplement: Figure 2—source data 1. [file elife-105017-fig2-data1.pdf]

Figure 2A

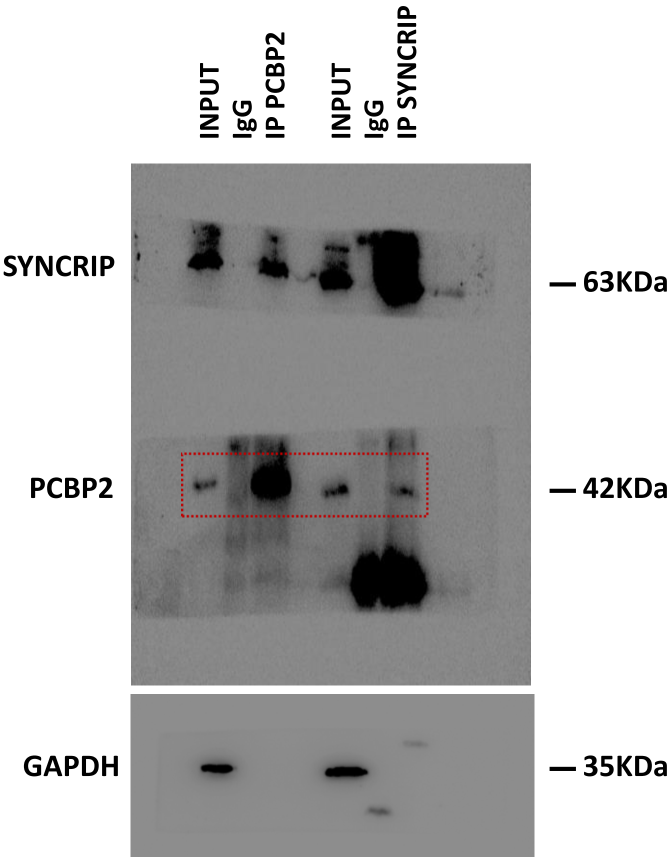

Figure 2B

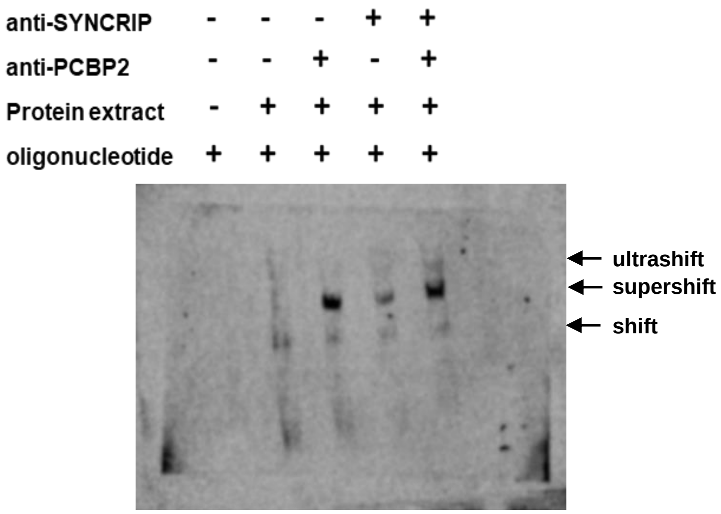

Figure 2C

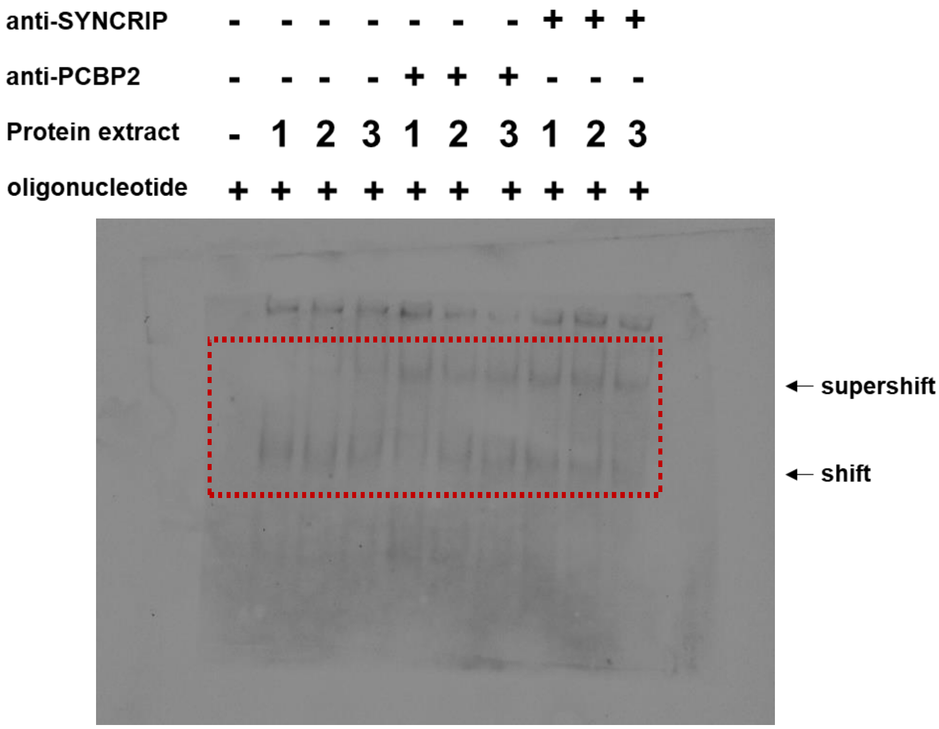

1: Protein extract from shCTR cells

2: Protein extract from shPCBP2 cells

3: Protein extract from shSYNCRIP cells
